# Exploring the impact of the COVID-19 pandemic on perceptual disturbances and dysfunctional eating attitudes and behaviors: A review of the literature

**DOI:** 10.3389/fpsyg.2023.1139261

**Published:** 2023-03-15

**Authors:** Johana Monthuy-Blanc, Giulia Corno, Sara Abou Chabake

**Affiliations:** ^1^Loricorps Research Unit, Centre de Recherche de l'Institut Universitaire en Santé Mentale de Montréal, Montréal, QC, Canada; ^2^Department of Sciences of Education, Université du Québec à Trois-Rivières, Trois-Rivières, QC, Canada; ^3^Department of Psychology and Psychoeducation, Laboratory of Cyberpsychology, Université du Québec en Outaouais, Gatineau, QC, Canada; ^4^Department of Psychology, LESPOIR Research Laboratory, Université de Montréal, Montréal, QC, Canada; ^5^Department of Psychology, TRAJETS Research Laboratory, Université de Montréal, Montréal, QC, Canada

**Keywords:** body image disturbances, low body esteem, weight and shape concerns, disordered eating, eating disorders, COVID-19, literature review

## Abstract

From the outbreak of the novel coronavirus 2019 (COVID-19) a new physical and social distancing environment has changed our lives and, more particularly, the way of perceiving oneself, as well as eating attitudes and behaviors. An increasing number of studies have highlighted a risky scenario in terms of negative perceptions of one’s body as well as disordered eating and eating disorder patterns in both clinical and general population. With regard to this postulate, this literature review posits two main concepts—perceptual disturbances and dysfunctional eating attitudes and behaviors—in the general and (sub-)clinical populations, to provide an understanding of these phenomena during the COVID-19 pandemic. The main objective of this article is to provide a comprehensive and critical review of published scientific literature about perceptual disturbances (i.e., negative body image, body image disturbances, low body esteem) and dysfunctional eating attitudes and behaviors, including disordered eating (e.g., restrictive eating, binge-eating episodes, overeating, emotional eating) and eating disorders features in community (i.e., general population) and clinical and sub-clinical samples worldwide during the COVID-19 pandemic. The PubMed, ScienceDirect, Ebsco, and Google Scholar databases were searched. The initial search produced 42 references. Scientific publications from March 2020 to April 2022 were included, and among the works compiled, only published research articles have been retained. Purely theoretical papers were also excluded. The final selection consisted of 21 studies, covering both community, clinical (i.e., eating disorder population), and sub-clinical samples. The details of the results are discussed taking into consideration the potential impact of changes in the way we perceive ourselves and interact with others (e.g., the popularity of videoconferencing and the over-use of social network sites due to social isolation) as well as changes in eating attitudes and behaviors, physical activity and exercise (e.g., as an emotional response to the insecurity generated by the pandemic context), in community and (sub-)clinical samples. The discussion sheds light on two outcomes: (1) a summary of findings with methodological considerations; (2) an intervention continuum to deal with the consequences of the COVID-19 pandemic; (3) and a final conclusion.

## 1. Introduction

In late 2019, the novel coronavirus disease 2019 (COVID-19) made its first appearance in Wuhan, China, and few months later, on March 11th, 2020, the World Health Organization declared that the COVID-19 crisis was a pandemic (World Health Organization, 2020). The COVID-19 pandemic has profoundly disrupted the lives of millions of people. Many countries across the world have implemented safety measures (e.g., quarantine, lockdown, travel restrictions, social distancing) to decrease the spread of the virus. These measures, although necessary to save lives and prevent severe cases of infection, have altered people’s daily lives on many levels, such as social, work, occupational, and leisure time. Such changes and challenges have encouraged researchers and healthcare professionals to investigate the impact of the pandemic situation on well-being and mental health (de Medeiros Carvalho et al., 2020; Salari et al., 2020; Torales et al., 2020; Vindegaard and Benros, 2020; Zhang et al., 2020).

Studies from the last 2 years have contended that self-perceptions have deteriorated during the COVID-19 pandemic, leading to distorted self-perceptions (Rodgers et al., 2020; Ahuja and Banerjee, 2021). A first threat comes from excessive social media consumption during the pandemic (Ahuja and Banerjee, 2021; Vall-Roqué et al., 2021). Since the first lockdown, social media have spread harmful and alarmist messages about overeating, weight gain, sedentary behavior (e.g., the hashtag “Quarantine15” or “COVID15,” originating from the phenomenon of “freshman-15” which refers to the belief that students tend to gain 15 lbs. during freshman year; “‘covibesity’,” the widespread rapid weight gain during lockdowns), and moralizing messages about staying healthy during the pandemic and practicing physical activity (Khan and Smith, 2020; Pearl, 2020; Ahuja and Banerjee, 2021; Lucibello et al., 2021; Schneider et al., 2022). For instance, people have been bombarded on social media with messages about the danger of being overweight, innumerable diets as well as fitness tutorials featuring ideal bodies. Such heightened pressure to stay and be healthy during COVID-19 times could have exacerbated concerns about weight and body shape, weight stigma leading to fatphobia, and could have increased perceptual disturbances such as body dissatisfaction (Ahuja and Banerjee, 2021; Schmid et al., 2022; Schneider et al., 2022). Psychological distress related to pandemic-related restrictions could also be associated with more negative body image perceptions and concerns (Ahuja and Banerjee, 2021; Swami et al., 2021; Schneider et al., 2022). Finally, stay-at-home, physical, and social distancing measures shifted many interactions to video-supported communications. Some authors have started to talk about the “Zoom effect” or the “Zoom dysmorphia disorder,” referring to the fact that continued (over)exposure to one’s own image could have led to “self-centric” thoughts and exacerbated the attention directed to one’s appearance, with a consequent negative impact on body image (Ahuja and Banerjee, 2021; Pikoos et al., 2021; Pino, 2022; Schneider et al., 2022; Weissman and Hay, 2022). Finally, the pandemic presented unique threats to what Bruch (1962) defined as perceptual disturbances. Conceptualized as a dimension of disturbances in perception, they have a negative effect on the ability to perceive, be aware of, and correctly interpret signals of the state of the body (such as negative body image, body image disturbances, low body esteem, etc.). At that time, perceptual disturbances were already related to a body shape that does not conform to the image accepted by society, therefore under enormous pressure and constant criticism (Bruch, 1974). Furthermore, perceptual disturbances are a well-established core of disordered eating behaviors and eating disorders (Stice et al., 1996, 1998; Cash and Deagle III, 1997; Burrows and Cooper, 2002; Stice, 2002, 2022; Fairburn et al., 2003; Neumark-Sztainer et al., 2003; Levine and Piran, 2004; Glashouwer et al., 2019; Andersen and Swami, 2021; Corno et al., 2022).

**Figure 1:**
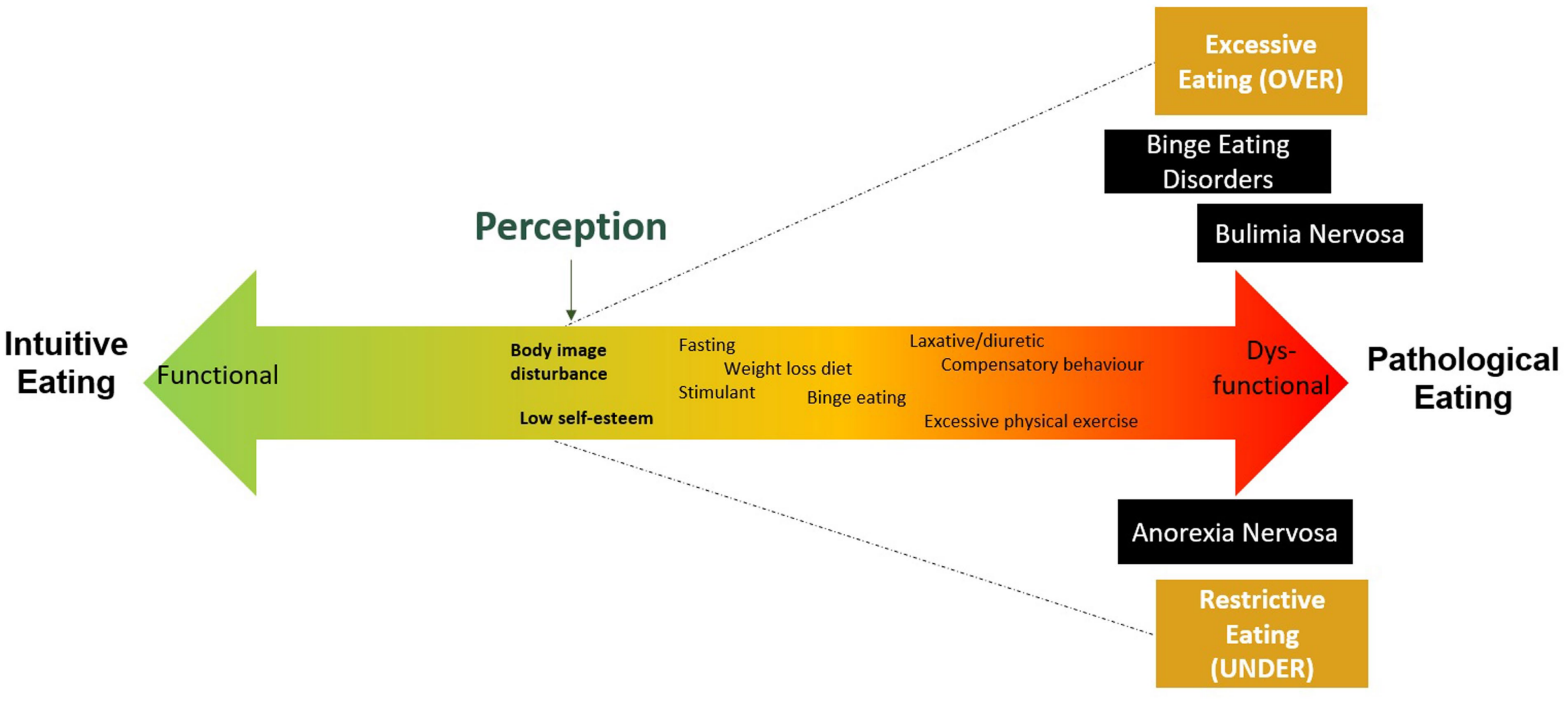
Dimensional approach.

Since the COVID-19 outbreak, experts on eating disorders cautioned about the detrimental impact of the pandemic situation on disordered eating habits and on eating disorder syndromes (Fernández-Aranda et al., 2020; Rodgers et al., 2020; Cooper et al., 2022). These considerations of eating features in the general and clinical population both led some authors to favor the dimensional mental health approach (Leung et al., 1996; Alvarenga et al., 2010; Sundgot-Borgen and Torstveit, 2010; Turgeon et al., 2015; Monthuy-Blanc et al., 2020). As presented in Figure 1, this holistic and dimensional approach proposes a conceptualization based on a continuum of severity ranging from a functional or asymptomatic state corresponding to intuitive eating (associated with food well-being), to dysfunctional or clinical state corresponding to pathological eating (eating disorders outlined in the Diagnostic and Statistical Manual of Mental Disorders, 5th Edition; DSM-5), through different, yet not clinical, eating attitudes and behaviors (e.g., emotional eating, restrictive eating, overeating; American Psychiatric Association, 2013; Tribole and Resch, 2020; Monthuy-Blanc et al., 2022).

This dimensional approach of dysfunctional eating attitudes and behaviors offers an alternative to categorical medical approaches which are limited in understanding the impact of the COVID-19 pandemic. Indeed, there are different ways in which the pandemic could have led to a new onset of dysfunctional eating behaviors in the general population. Public health mitigation strategies could have limited opportunities for regular food shopping, resulting in changes in food consumption and eating habits. On one hand, threats of food shortages and perceived body shape and weight gain could have given rise to restrictive eating behaviors associated to over/excessive exercising (Schlegl et al., 2020; Termorshuizen et al., 2020; Nisticò et al., 2021; Miskovic-Wheatley et al., 2022). On the other hand, accumulation of food stored up and stay-at-home orders could have increased the risk of overeating and binge eating episodes (Di Renzo et al., 2020; Scarmozzino and Visioli, 2020; Schlegl et al., 2020; Touyz et al., 2020; Weissman et al., 2020; Corno et al., 2022; Weissman and Hay, 2022). Furthermore, stress and emotional reactions to the pandemic situation could have contributed to dysfunctional eating attitudes. For instance, emotional eating could have been adopted as a dysfunctional coping mechanism to overcome pandemic-related stress and negative emotions (Ferrell et al., 2020; Madalı et al., 2021; Corno et al., 2022; Sanlier et al., 2022). Life during the COVID-19 crisis could have been particularly challenging for people living with severe eating disorders or with a history of eating disorders, leading to a deterioration of symptoms and relapses (Fernández-Aranda et al., 2020; Touyz et al., 2020; Weissman et al., 2020; Linardon et al., 2021; Todisco and Donini, 2021; Devoe et al., 2022). Difficulties in regulating emotions, increased anxiety and depression, food insecurity, uncertainty about the future, disrupted access to mental health services (e.g., limited access to outpatient services and day treatment), changes in routines (e.g., attending school and work) and physical activity as well as social isolation could have had a negative impact on the lives of people living with dysfunctional eating attitudes and behaviors (Brown et al., 2021; Coulthard et al., 2021; Linardon et al., 2021; Vuillier et al., 2021; Devoe et al., 2022; Gao et al., 2022; Haghshomar et al., 2022; Schneider et al., 2022). Indeed, in their qualitative study, Brown et al. (2021) reported that lockdowns periods were particularly challenging for their participants (i.e., 10 adults with eating disorders), since social isolation and the lack of social scrutiny led them to go in search of comfort in familiar behaviors such as eating restraint or binge eating.

**Figure 2:**
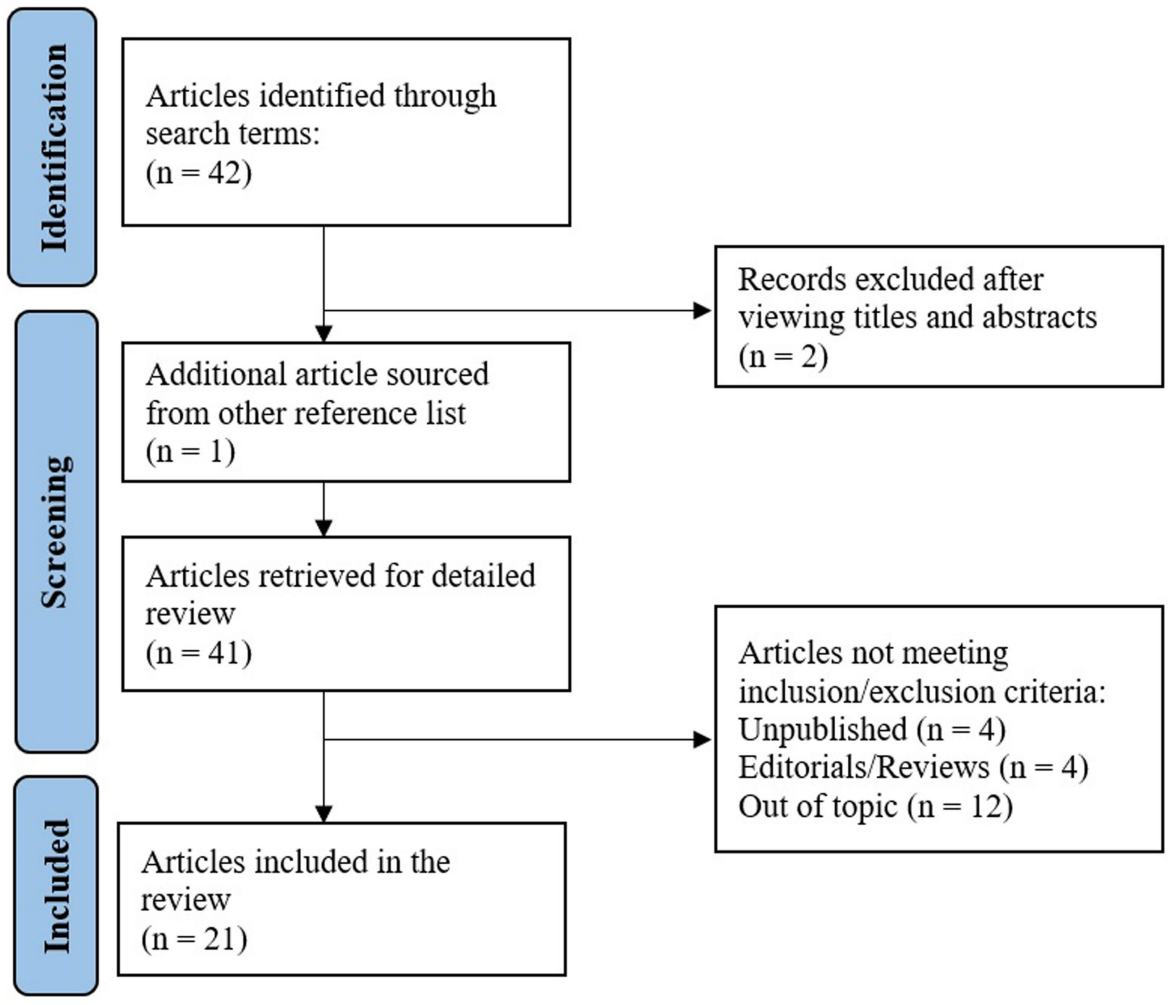
Flow chart of the selection of reviewed articles.

The main objective of this article is to provide a comprehensive and critical review of published scientific literature about perceptual disturbances (i.e., negative body image, body image disturbances, low body esteem) and dysfunctional eating attitudes and behaviors, including disordered eating (e.g., restrictive eating, binge eating episodes, overeating, emotional eating) and eating disorders features in community (i.e., general population), sub-clinical and clinical samples worldwide during the COVID-19 pandemic. The PubMed, ScienceDirect, Ebsco, and Google Scholar databases were searched. The terms used and Boolean entries were: (disordered eating OR eating disorders OR unhealthy eating OR dysfunctional eating behaviors) AND (body image OR body perception OR body esteem OR dysmorphia) AND (COVID-19 OR coronavirus OR 2019-ncov OR sars-cov-2 OR cov-19). The initial search produced 42 references (see Figure 2). Scientific publications from March 2020 (i.e., from the beginning of the COVID pandemic context for most countries) to April 2022 were included. Among the works compiled, only published research articles have been retained. Purely theoretical papers were excluded, and since obesity is not considered to be an eating disorder, publications specifically targeting people living with obesity were also excluded. Regarding data collection, specific time-frame criteria were not considered as an inclusion criterion for the selection of the publications. However, an evaluation of the publications’ methodological strength regarding the COVID-19 specification is proposed in the discussion. The final selection consisted of 21 studies, 18 covering the general population sample and 3 (sub-)clinical samples. Tables 1, 2 summarize the studies reviewed about perceptual disturbances, dysfunctional eating attitudes and behaviors in community and (sub-)clinical samples.

**Table 1:** Studies about perceptual disturbances and dysfunctional eating attitudes and behaviors in community samples during COVID-19.

Author(s), year, country	Methodology/study design	*N* (total) (sex at birth)	Measures	Outcomes
Baceviciene and Jankauskiene (2021) LT	QUAN/observational study design (pre- vs. post-lockdown)	Pre 1850 Post 230 students (male & female)	SATAQ-4, EDE-Q, MBSRQ-AS, LTEQ, WHOQOL-BREF, RSES, self-reported dietary habits, sleep duration, time browsing internet, binge drinking, health	PD: No negative body image increases; increase in thin internalization; among female participants: increase in media pressure to thin/low fat ideals and increased appearance evaluation DEAB: Decrease in unhealthy eating habits
Buckley et al. (2021) AU	QUAN&QUAL/cross-sectional	162 athletes (male and female)	EAT-26, 2 quantitative self-reported questions on change in body image and food relationship, 4 qualitative questions on changes in disordered eating related to COVID-19	PD: 34.8% worsened body image (fear of body composition changes, fear of anticipatory body changes, fear of weight gain, increased comparison to others through social media); changes in exercise were the most significant factor that influenced their body perception DEAB: 32.8% worsened food relationship (increased binges, restraint, guilt, shame, increase/decreased control over food); changes in exercise were the most significant factor that influenced their relationship with food
Corno et al. (2022) CAN	QUAN/cross-sectional	161 (female)	Self-rated changes in the frequency of disordered eating behaviors, EDE-Q-WC, EDE-Q-SC, body dissatisfaction (*e*LoriCorps-IBRS 1.0)	PB: No clinical levels of weight and body shape concerns; presence of body image dissatisfaction DEAB: Increase of the frequency of all disordered eating behaviors (restrictive eating, overeating, emotional eating); high weight concerns predicted increased restrictive eating; higher body image dissatisfaction predicted increased emotional eating
Costa et al. (2022) BR	QUAN/cross-sectional	598 (male and female)	TFEQ-R21, FFQ, self-reported perceptions of eating habits, and life habits during the pandemic, self-reported perception on body image satisfaction	PD: Association between cognitive restraint and perception of being overweight; association between emotional eating and body dissatisfaction, perception of overweight; association between uncontrolled eating and body dissatisfaction DEAB: Association between cognitive restraint and being active, attempted weight loss, better eating habits, increased consumption of refined cereals and fast foods; association between emotional eating and increased stress, worse sleep, attempted weight loss; increased consumption of amount of food; increased food delivery purchase; increase in consumption of sweets and desserts; decreased consumption of vegetables; association between uncontrolled eating and working (> 8 h/day), increased stress, worse eating habits, increased consumption of amount of food
Czepczor-Bernat et al. (2021) PL	QUAN/cross-sectional	1,354 (female)	CDRS, EOQ, EMS, MEQ, COVID-19-related stress measure	PD: 4 clusters as (World Health Organization, 2020) healthy body weight, no covid-related stress and high body image disturbances (de Medeiros Carvalho et al., 2020) overweight, no COVID-related stress, and high body image disturbances (Salari et al., 2020) healthy body weight, COVID-related stress, and low body image disturbances (Torales et al., 2020) overweight, COVID-related stress, and high body image disturbances DEAB: Women of cluster 4 presents a risk pattern of disordered eating; Body weight status, COVID-19-related stress, and BD, may contribute to the intensity of disordered eating
Faramarzi et al. (2021) IR	QUAN/ cross-sectional study	535 high school students (female)	MBSRQ; BPAQ, EDDS	PD: Significant positive correlations between BI and PA; No significant correlation between BI and BMI DEAB: No significant correlations between BI and AN, BN, and BED; no relationships were observed between PA and AN, BN, and BED
Flaudias et al. (2020) FR	QUAN/cross-sectional	5,738 students (male and female)	HADS, PSS-10, EDI-2, self-report lockdown-related stress	PD: High BD in this study was a risk factor for binge eating and dietary restriction. DEAB: Stress related to lockdown, probable eating disorder and greater anxiety were associated with greater likelihood of binge eating and dietary restriction; higher BMI and greater levels of depression were associated with binge eating; underweight, obesity, and greater exposure to COVID-19 related media was associated with increased eating restriction
Gobin et al. (2021) CA	QUAN/cross-sectional	143 (female)	EHQ, Self-report changes in eating, exercise, and social media habits during the pandemic	PD: Lockdown worsened various aspects of body image among women with high ON tendencies: they felt that they have gained weight, and an increased pressure to lose weight. Social media pressure to lose weight and exercise worsened for women high on ON tendencies DEAB: Women high on ON tendencies reported eating a lot more food and thin more about food
Gullo and Walker (2021) US	QUAN/cross-sectional	143 (male, female, trans-male and non-binary)	DASS, MBSRQ, BES	PD: Physical appearance satisfaction and appearance orientation decreased after lockdown; self-viewing time did not predict appearance orientation and satisfaction DEAB: Binge eating did not increase following lockdowns; self-viewing time did not predict binge eating
Haddad et al. (2020) LB	QUAN/cross-sectional	407 (male and female), general population and dietitian clients group	Self-reported quarantine and confinement stressors, fear of COVID-19, BPS-A, EDE-Q	PD: Anxiety and higher fear of COVID-19, higher BMI, practicing physical exercise, and a higher number of adults living in the quarantine/confinement were associated with higher body shape and weight concerns. DEAB: Greater fear of COVID-19 was associated with higher eating restraint.
Haddad et al. (2021) LB	QUAN/cross-sectional	407 (male and female)	Self-reported current fear of COVID-19, SBPS, LAS, BPS-A, EDE-Q, PAI	PD: Perceived weight change was found in 52.1%, while there was no significant variation of BMI; higher fear of COVID-19 and higher self-reported weight change were associated with lower weight change perception DEAB: Higher eating concerns and anxiety, and longer confinement were significantly associated with higher perceptions in weight change
Jackson et al. (2022) US	QUAN/longitudinal	288 (male and female)	BSQ-8, FAS, BAS-2, DSQ, TFEQ-R18, IES-2	PD: Negative BI might decrease body-food choice congruence and thus decrease diet quality; positive body image might increase body-food choice congruence, which promotes diet quality; body image was not directly associated with diet quality DEAB: Maladaptive eating behaviors were not associated with diet quality
Mannino et al. (2021) IT	QUAN/longitudinal	115 (male and female)	FC-19S, EDE-Q, MFU, APUF, Information about quarantine	PD: Maladaptive Facebook use mediated the relationship between daily time on Facebook and shape concerns; Shape and weight concerns increased during the pandemic DEAB: Increased eating concerns; a direct positive relation between daily time on Facebook and eating concerns was found; higher fear of COVID-19 was positively associated to time spent on Facebook, which in turn predicted disordered eating cognitions
Pineda-García et al. (2021) MX	QUAN/cross-sectional	276 (male and female)	GAS, EAT-26, body dissatisfaction (FRS), self-reported labor status and degree of confinement	PD: Anxiety increased BD DEAB: Increased anxiety and BD led to increased behavior
Quathamer and Joy (2022) CA	QUAL	70 self-identifying LGBTQ + individuals (male and female)	Open-ended questions related to COVID-19, Semi-structured virtual interviews	PD: Competing social discourses created complex and often contradictory meanings of bodies and BI DEAB: COVID-19-related self-isolation was a reprieve from constant body monitoring for some, and a time of continued pressure to embody body ideals for others
Robertson et al. (2021) UK	QUAN/cross-sectional	264 (male and female)	PHQ-4, mental health and developmental disorder, self-perceived changes in eating, exercise, and body image during the lockdown	PD: Women were more likely than men to report worsening BI; those with a current or past diagnosis of EDs reported significantly greater behaviors and concern about physical appearance; great self-perceived changes in body image was associated with higher psychological distress DEAB: Women were more likely than men to report increasing struggles with regulating eating, and preoccupation with food; those with a current or past diagnosis of EDs reported significantly greater difficulties in regulating eating, increased preoccupation with food, and exercise thoughts; great self-perceived changes in eating was associated with higher psychological distress
Sajedi et al. (2021) TR	QUAN/Descriptive and correlational	181athletes (male and female)	CDAS, MBIQ, EDQ	PD: BI has a significant negative effect on ED DEAB: Corona anxiety had a significant positive effect on ED
Trott et al. (2021) US	QUAN/Longitudinal	319 health club users (male and female)	EAI, EAT-26, BDDQ, self-reported time spent to exercise for leisure, current lockdown	PD: No differences in body dysmorphic disorder tendencies DEAB: Higher ED symptomology post-COVID-19 lockdown Other: Increased leisure-time exercise post-COVID-19 lockdown; Lower exercise addiction scores post-COVID-19 lockdown

PD: Perceptual disturbances; DEAB: dysfunctional eating attitudes and behaviors; QUAN: quantitative design; QUAL: qualitative design; PA: physical activity; ED: eating disorder; BI: body image; BD: body dissatisfaction; AN: anorexia nervosa; BN: bulimia nervosa; BED: binge eating disorder; SATAQ-4: Sociocultural Attitudes Towards Appearance Questionnaire; MBSRQ-AS: Multidimensional Body–Self Relations Questionnaire–Appearance Scale; EDE-Q: Eating Disorder Examination Questionnaire; LTEQ: Leisure Time Exercise Questionnaire; WHOQOL-BREF: World Health Organization Quality of Life-BREF Questionnaire; RSES: Rosenberg’s Self-Esteem Scale; EAT-26: Eating Attitudes Test-26; EDE-Q-WC: Eating Disorder Examination Questionnaire—weight concerns subscale; EDE-Q-SC: Eating Disorder Examination Questionnaire—shape concerns subscale; eLoriCorps IBRS 1.0: eLoriCorps Immersive Body Rating Scale 1.0; TFEQ-R21: Three-factor eating questionnaire; FFQ: Food frequency questionnaire; CDRS: Contour Drawing Rating Scale; EOQ: Emotional Overeating Questionnaire; EMS: Eating Motivation Survey; MEQ: Mindful Eating Questionnaire; BIQLI: Body Image Quality of Life Inventory; SIBID-S: Short form of the Situational Inventory of Body-Image Dysphoria; BDI-II: Beck Depression Inventory-II; MBSRQ: Multidimensional Body–Self Relations Questionnaire; BPAQ: Baecke’s Physical Activity Questionnaire, EDDS: Eating Disorder Diagnostic Scale; HADS: Hospital Anxiety and Depression Scale; PSS-10: Perceived Stress Scale; EDI-2: Eating Disorder Inventory 2nd edition; EHQ: Eating Habits Questionnaire; DASS: Depression Anxiety Stress Scale; BES: Binge Eating Scale; SBPS: Short Boredom Proneness Scale; LAS: Lebanese Anxiety Scale; BPS-A: Buss-Perry Scale – Anger subscale; PAI: Physical Activity Index; BSQ-8: Body Shape Questionnaire; FAS: functionality appreciation scale; BAS-2: Body Appreciation Scale; DSQ: Dietary screener questionnaire; TFEQ-R18: Three Factor Eating Questionnaire; IES-2: Intuitive eating; FC-19S: Fear of COVID-19 Scale; MFU: Maladaptive Facebook Usage; APUF: Active and Passive Facebook Use Scale; GAS: Goldberg Anxiety Scale; PHQ-4: FRS: Figure rating scale; Patient Health Questionnaire; CDAS: Corona Disease Anxiety Scale; MBIQ: Mental Body Image Questionnaire; EDQ: Eating Disorders Questionnaire; EAI: Exercise Addiction Inventory; BDDQ: Body Dysmorphic Disorder Questionnaire.

**Table 2:** Studies about perceptual disturbances and dysfunctional eating attitudes and behaviors in ED-clinical and sub-clinical sample.

Author(s), year, country	Methodology/study design	*N* (total) (sex at birth)	Measures	Summary of findings
Brownstone et al. (2022) N/A	QUAL/ Inductive codebook thematic analysis	31 sub-clinical individuals (male and female)	Semi-structured interview on COVID-19 experiences	PD: All participants expressed a heightened sense of surveillance of their bodies, frequently paired with body dissatisfaction, during COVID-19; A few participants ruminated on how others might perceive changes to their body once social distancing measures were released; Participants remarked that eating and exercising, daily habitual behaviors, were experientially impacted by how much or little they might be seen and witnessed by others. DEAB: Relationships between increased disordered eating, anxiety, stress, and food insecurity
Savarese et al. (2022) IT	QUAN/ pilot	14 patients (female)	EDI-3, BU, DS–R	PD: 88.2% passed the clinical cut-off on the general psychological maladaptation scale showing widespread dissatisfaction with BI DEAB: 30% of the patients reported the onset of symptoms for anorexia nervosa during the lockdown; Increased diet related stress
Zhou and Wade (2021) AU	QUAN/ longitudinal (pre- vs. post-lockdown)	100 students at risk of developing ED (female)	EDE-Q, BI-AAQ, SCS-SF, FSCS, PANAS	PD: Increase in BI flexibility over time DEAB: Significant increase in weight concerns, disordered eating, and negative affect during the COVID-19 compared to pre-COVID-19

Country: N/A: Non-applicable. Methodology: Quanti: quantitative; Quali: qualitative. Measures: EDI-3: Eating Disorder Inventory-3; BUT: Body Uneasiness Test; DS-R: Disgust Scale-Revised; EDE-Q: Eating Disorder Examination Questionnaire; BI-AAQ: Body Image Acceptance & Action Questionnaire; SCS-SF: Self-Compassion Scale – Short Form; FSCS: Fear of Self-Compassion Scale; PANAS: Positive and Negative Affect Schedule.

## 2. Studies on the impact of the COVID-19 pandemic context in community and (sub-)clinical samples

According to the objective of the current review, two types of outcomes were highlighted: (World Health Organization, 2020) perceptual disturbances (i.e., negative body image, body image disturbances, low body esteem) and (de Medeiros Carvalho et al., 2020) dysfunctional eating attitudes and behaviors (i.e., from disordered eating to eating disorder features). Overall, the studies of this comprehensive and critical literature review show an increase in perceptual disturbances and dysfunctional eating attitudes and behaviors in community and (sub-)clinical samples beyond a variability of outcomes.

### 2.1. Community sample

The first scientific publications about perceptual disturbances and dysfunctional eating attitudes and behaviors date back to September 2020, when many countries around the world started to face the second wave of the pandemic. In this review, only two studies published in 2020 provided data about the beginning of the COVID-19 pandemic, whereas the majority of studies (*n* = 11) were published in 2021 during different waves of the COVID-19 pandemic. Four studies were published recently in 2022. Studies are presented in the following paragraphs in chronological order (i.e., in terms of publication date) and by geographic area. A resume of the community sample-based studies included in this review is available in Table 1.

Flaudias et al. (2020) conducted a first cross-sectional study with a sample of undergraduate students (*N* = 5,738) during the first week of lockdown in France. The authors found that higher body dissatisfaction was associated to a higher probability of reporting binge eating episodes and dietary restriction. Furthermore, stress related to confinement, probable eating disorders, as well as greater anxiety were associated with a greater likelihood of binge eating and dietary restriction. A higher self-reported body mass index (BMI) and greater depression were associated only to an increased probability of binge eating behaviors. Finally, being underweight, obesity and greater exposure to COVID-19 related media was associated with increased eating restriction (Flaudias et al., 2020). Haddad et al. (2020) conducted a study in Lebanon at the beginning of the COVID-19 outbreak (i.e., beginning of April 2020). They investigated the relation between confinement stressors, eating behaviors and negative body image among the general population and a population of adults attending diet clinics (*N* = 407). The authors found that a greater fear of COVID-19, a higher self-reported BMI and excessive physical exercise were associated with both greater eating restraints and shape and weight concerns. Higher anxiety and a greater number of people living in confinement were associated only to stronger shape and weight concerns. When comparing the two samples, the authors found that fear of COVID-19 was correlated with eating, shape, and weight concerns, more specifically in the dietitian clients group (Haddad et al., 2020).

Four studies in 2021 were conducted in the European region. In Lithuania, Baceviciene and Jankauskiene, (2021) investigated university students’ attitudes towards appearance, body image, eating attitudes and behaviors, physical activity, and quality of life during the pandemic (*N* = 1850). Their study showed a significant increase in thin internalization, and a decrease of unhealthy eating habits among students of both genders. In women, the authors found a significant self-reported increase of media pressure towards thin/low fat ideals but also an increase in appearance evaluation (Baceviciene and Jankauskiene, 2021). Czepczor-Bernat et al. (2021) conducted a cross-sectional study in Poland from January to March 2021. With their cluster-analysis, the authors revealed the presence of a group of women with a risky pattern of disordered eating behaviors (*N* = 1,354). Specifically, overweight women, with high body dissatisfaction and COVID-related stress, presented higher levels of disordered eating (e.g., emotional overeating, affect regulation motive for eating; Czepczor-Bernat et al., 2021). In a longitudinal study conducted in Italy (T0: first lockdown in Italy, March–April 2020; T1: time of eased restriction, June 2020) among members of a Facebook online community focused on eating disorders (*N* = 115), Mannino et al. (2021) found that a greater fear of COVID-19 during the first lockdown was associated with self-reported time spent on Facebook, which in turn predicted disordered eating cognitions after 2 months. Furthermore, the authors found that maladaptive Facebook use (i.e., engaging in negative social comparisons on Facebook) mediated the relation between time spent on Facebook and participants’ body shape concerns (Mannino et al., 2021). Robertson et al. (2021) conducted a cross-sectional study (*N* = 264) exploring changes in body image, eating and exercise behaviors during the 2020 summer lockdown in the United Kingdom (May–June 2020). The authors found three key results: first, women were more likely to report more difficulties in regulating eating, having more preoccupation around food and physical appearance. Secondly, participants who reported a current or past eating disorder, were more preoccupied with food, their physical appearance and have increased thoughts about exercise. Finally, elevated rates of perceived changes in body image and eating were associated to high psychological distress (Robertson et al., 2021).

In 2021, two studies have reported results from two countries in the Middle East. The objective of the study conducted by Sajedi et al. (Sajedi et al., 2021) in Turkey, was to examine the impact of body image and anxiety related to COVID-19 on athletes’ eating disorder symptomatology (*N* = 181). The authors found that positive body image (e.g., body image satisfaction, positive attitudes about weight) had a significant negative effect on eating disorder symptomatology, whereas COVID-19 related anxiety had a positive effect on eating disorder symptoms (Sajedi et al., 2021). Haddad et al. (2021) investigated in Lebanon the relation between COVID-19 confinement-related variables and the perception of weight changes (*N* = 407). The authors found that no variation in BMI was detected, even if participants perceived a weight change from before to after the confinement in April 2020. Furthermore, higher fear related to the COVID-19 virus and greater weight change were associated with lower weight change perception. On the contrary, greater eating concerns, anxiety and longer confinement were associated with higher weight change perception (Haddad et al., 2021).

Fours studies have reported findings from North America and one study from Central America in 2021. Among the North American studies, two of them were conducted in Canada. Gobin et al. (2021) explored changes to adult women’s body image, eating and social media habits during a lockdown in Canada (May–June 2020). More specifically they compared women with and without signs of orthorexia nervosa (*N* = 143). Orthorexia nervosa can be defined as a pathological obsession with healthy food accompanied by a restrictive diet, complex ritualized patterns of eating and avoidance of certain categories of food (e.g., meet, diary) and/or ingredients (e.g., sugar) that are considered as impure/unhealthy (Koven and Abry, 2015; Gobin et al., 2021). Although orthorexia nervosa is often associated with significant impairment, it is not yet classified as an eating disorder in the DSM-5. For this reason, the study of Gobin et al. (2021) was not included in Table 2. The results of this study suggest that women with high tendencies of orthorexia nervosa experienced an exacerbation of disordered eating thoughts (i.e., thinking more about food) and behaviors (i.e., including eating a lot more food, and dieting behaviors), a worsening of body image (i.e., feelings of having gained a lot of weight and increased pressure to lose weight), and perceiving more pressure from social media to lose weight and exercise during the lockdown (Gobin et al., 2021). The second study published in 2021 and conducted in Canada is by Quathamer and Joy (2022). These authors conducted a qualitative study among the LGBTQ + community (*N* = 70) during the second wave of 2020. Pandemic restrictions had complex and often contradictory consequences among the participants. Stay-at-home orders and self-isolation were a negative experience for some participants in terms of impact on body image and eating habits. They reported that the COVID-19 restrictions reinforced disordered eating and body surveillance. Conversely, other participants reported that this period was useful to critically reflect on their experiences and embrace new ways of viewing and considering their body (Quathamer and Joy, 2022). Gullo and Walker (2021) investigated whether self-viewing time during lockdown periods in the US (February 2020: retrospective recall; April 2020), could be associated with deteriorated mood, binge eating and body image (*N* = 143). The authors found that physical appearance orientation and satisfaction decreased, whereas binge eating did not increase after the lockdowns. Contrary to the authors’ hypotheses, self-viewing time did not predict binge eating, physical appearance satisfaction and orientation (Gullo and Walker, 2021). Trott et al. (2021) conducted a longitudinal study to evaluate exercise addiction, leisure time exercise, eating disorder symptomatology, and body dysmorphic disorder tendencies among health club users during lockdowns (*N* = 319). Exercise addiction decreased, whereas leisure time exercise increased post-lockdown. Eating disorder symptomatology significantly increased post-lockdown whereas no differences were found in terms of body dysmorphic disorder tendencies (Trott et al., 2021). In a cross-sectional study conducted among Mexican adults (*N* = 276), Pineda-García et al. (2021) investigated the relationship between labor status, confinement due to COVID-19, body image dissatisfaction and anxiety, as well as explored the effects of these phenomena on bulimic behavior. The authors found that anxiety increased body image dissatisfaction, whereby increased anxiety and body dissatisfaction directly led to increased bulimic behavior (Pineda-García et al., 2021).

Finally, in 2021 from Oceania, Buckley et al. (2021) published the results of their convergent mixed-method study aimed at investigating whether current and former athletes (*N* = 162) experienced a deterioration in their body image, relationship with food and eating disorder psychopathology at the beginning of the pandemic in Australia, from April to May 2020. Twenty-one percent of their participants presented an eating disorder psychopathology. Notice that this study was not included in Table 2 because of this low percentage of eating disorders population. The body image and food relationship had worsened in more than 30% of participants. The deterioration in the relationship with their body emerged in terms of fear of body composition changes, fear of anticipatory body changes, fear of weight gain, and increased comparison to others through social media. Changes related to the body were perceived as a loss of identity and sense of self (“body grief”). Increased preoccupation over the body was linked to an attempt to control food, which in turn increased body preoccupation. This self-perpetuating cycle was related to restricting/binging behaviors and a worsened relationship with food (Buckley et al., 2021).

Four studies have been published in 2022, two of which were conducted in North America, one in South America and one in the Middle East. Corno et al. (2022) conducted a cross-sectional study among Canadian women (*N* = 161) between October 2020 and May 2021. The authors aimed to explore changes in negative body image and in the frequency of disordered eating behaviors, as well as investigate the possible relation between negative body image and changes in dysfunctional eating behaviors. The results of this study revealed that women reported clinical level of weight and shape concerns and that they were dissatisfied with their body image. Regarding disordered eating behaviors, the authors found that restrictive eating, overeating and emotional eating behaviors increased since the pandemic outbreak. Furthermore, higher weight concerns predicted an increase in the frequency of restrictive eating, while higher body image dissatisfaction predicted a rise in emotional eating (Corno et al., 2022). Jackson et al. (2022) conducted a longitudinal study in the United States from July to August 2020. In their study, the authors seek to understand the relationship between negative and positive body image, adaptive and maladaptive eating, and diet quality among a community sample of adults (*N* = 288). The results showed that body image and maladaptive eating did not have a direct effect on diet quality. However, positive body image led to an increase in body-food choice congruence (i.e., the degree of congruence between food choices and the needs of the body), which in turn increased diet quality. Conversely, negative body image decreased body-food choice, thus decreasing diet quality (Jackson et al., 2022). Costa et al. (2022) conducted a cross-sectional study in Brazil between December 2020 and January 2021, with the objective to examine the association between cognitive restraint, emotional eating, uncontrolled eating, body image-related factors, eating and life habits, and food consumption. Among the sample (*N* = 598), cognitive restraint was the dimension of eating behavior presenting the highest mean. Regarding eating habits, cognitive restraint was associated with better eating habits, decreased consumption of fast-foods and refined cereals. Regarding body image-related factors, cognitive restraint was associated with the perception of being overweight. Cognitive restraint was also associated with having tried to lose weight and being more active. Emotional eating was associated with an increased amount of food consumed, increased use of food delivery, and eating fewer vegetables. Furthermore, emotional eating was associated with the perception of being overweight and attempts to lose weight. Finally, uncontrolled eating was associated with worse eating habits, an increased amount of food consumed and body dissatisfaction (Costa et al., 2022). Faramarzi et al. (2021) conducted a cross-sectional study among female high school students in Iran (*N* = 535). Their objective was to examine the relation between physical activity, body image and eating disorder symptomatology in the context of the COVID-19 pandemic. The results of this study highlighted a positive relation between physical activity and body image. Eating disorder symptomatology was not related to body image nor to physical activity (Faramarzi et al., 2021).

### 2.2. Eating disorders (sub-)clinical samples

Notice that only one study was conducted in 2020 during the first year of the COVID-19 pandemic (Zhou and Wade, 2021). Two studies were published in 2022 (Brownstone et al., 2022; Savarese et al., 2022) and only one study included a clinical sample of patients with eating disorders (Savarese et al., 2022). Table 2 resumes studies conducted among clinical and sub-clinical samples.

Zhou and Wade (2021) conducted a quantitative longitudinal study pre- and post-lockdown among female students at risk of developing eating disorders (*N* = 100). Forty-one participants were included from September 2019 until March 2020 (pre-pandemic) and 59 followed after physical distancing began during the pandemic. In their study, the authors sought to understand female students’ risk of developing eating disorders and the effectiveness of online interventions during the COVID-19 pandemic. The results showed that participants experienced increased levels of disordered eating after the onset of COVID-19. Weight concerns, disordered eating and negative effects significantly increased during the pandemic compared to pre-lockdown. In contrast, there was an increase in body image flexibility over time. For the second study, Brownstone et al. (2022) conducted a qualitative study among a sub-clinical sample of Black, Indigenous, and People of Color (BIPOC; *N* = 31) during COVID-19 using a semi-structured interview on COVID-19 experiences. The authors used an inductive codebook thematic analysis and showed that most of the participants expressed fear of others’ perceptions on their bodies after lockdown, paired with increased body dissatisfaction. They also revealed an increase in disordered eating and eating disorders symptoms related to anxiety, stress, and food insecurity. Five themes were identified: body surveillance and dissatisfaction, movement and intake fixation, food scarcity and resource concerns, visible changes to the body and eating habits, and body vulnerability. Changes to the body and eating experiences were characterized by a continuum of feelings between distress to resilience. Savarese et al. (2022) conducted a pilot study between April and June 2021 using a sample of women with clinical anorexia nervosa (*N* = 14). The aim of this study was to evaluate anorexia nervosa symptoms, body image and the relationship between the onset of symptom during the COVID-19 pandemic. The results revealed an increase in eating disorder symptoms. Indeed, 30% of the females interviewed reported the onset of anorexia nervosa symptoms during the lockdown. Moreover, 88.2% of the sample passed the cut-off on the general psychological maladaptation scale showing widespread body dissatisfaction and increased diet-related stress.

## 3. Discussion

### 3.1. Methodological considerations (general and specific to COVID-19)

According to methodological considerations, this review includes studies with variability in how individuals respond to COVID-19, as do other reviews (Ahuja and Banerjee, 2021; Linardon et al., 2021; Miniati et al., 2021; Monteleone et al., 2021; Sideli et al., 2021; Devoe et al., 2022; Gao et al., 2022; Haghshomar et al., 2022; Schafer et al., 2022; Schneider et al., 2022). This postulate generates a discussion about general and COVID-19-specific methodological considerations. Regarding general methodological considerations, 18 studies were purely quantitative, 1 adopted a mixed-methods design, and 2 were qualitative. Among 19 studies that reported quantitative findings, 12 were cross-sectional, 4 were longitudinal, and 1 was a cohort study. Among 3 studies that reported qualitative findings, 1 used only online surveys and 2 used a combination of online surveys and semi-structured interviews. Six studies reported predictive models. The qualitative studies analyzed the data using thematic analysis (*n* = 1), discourse analysis (*n* = 1) and content analysis (*n* = 1). The included studies were conducted in more than 13 countries, predominantly in Europe and North America (United States *n* = 3, Mexico *n* = 1, Canada *n* = 3, United Kingdom *n* = 1, Italy *n* = 2, Lithuania *n* = 1, Poland *n* = 1, France *n* = 1, Turkey *n* = 1, Iran *n* = 1, Lebanon *n* = 2, Brazil *n* = 1 and Australia *n* = 2). All studies were conducted with adults. Less than half of the included studies (*n* = 9) reported participants’ ethnicity and/or racialized group belonging, and only two studies provided information regarding participants’ sexual orientation.

Regarding specific COVID-19 methodological considerations, due to the novelty of COVID-19 and the need to gain a comprehensive understanding of perceptual disorders and dysfunctional eating attitudes and behaviors during the pandemic, a “COVID-19 adequacy mention” has been proposed (see Table 3).

**Table 3:** Studies about COVID-19 adequacy in community and (sub-)clinical samples.

	Author(s), year, country	Sample	Adequacy			Findings	
			L	M	H	PD	DEAB
1	Baceviciene and Jankauskiene (2021)	Community		x		A	Hi
2	Buckley et al. (2021)	Community			x	He	He
3	Brownstone et al. (2022)	(Sub-)Clinical			x	He	He
4	Corno et al. (2022)	Community			x	He	He
5	Costa et al. (2022)	Community			x	He	A
6	Czepczor-Bernat et al. (2021)	Community			x	He	He
7	Faramarzi et al. (2021)	Community	x			A	A
8	Flaudias et al. (2020)	Community	x			He	He
9	Gobin et al. (2021)	Community		x		He	n.a
10	Gullo and Walker (2021)	Community		x		He	He
11	Haddad et al. (2020)	Community			x	He	He
12	Haddad et al. (2021)	Community			x	He	He
13	Jackson et al. (2022)	Community	x			He	A
14	Mannino et al. (2021)	Community			x	He	He
15	Pineda-García et al. (2021)	Community		x		He	He
16	Quathamer and Joy (2022)	Community			x	He	He
17	Robertson et al. (2021)	Community			x	He	He
18	Sajedi et al. (2021)	Community		x		He	He
19	Savarese et al. (2022)	(Sub-)Clinical	x			He	He
20	Trott et al. (2021)	Community		x		Hc	He
21	Zhou and Wade (2021)	(Sub-)Clinical		x		Hi	He

L: Low COVID-19-related adequacy; M: Moderate COVID-19-related adequacy; H: High COVID-19-related adequacy; A: ambiguity of findings on perceptual disorders and dysfunctional eating attitudes and behaviors; He: homogeneity of findings for an exacerbation of perceptual disorders and dysfunctional eating attitudes and behaviors; Hi: homogeneity of findings for an improvement of perceptual disorders and dysfunctional eating attitudes and behaviors; Uc: homogeneity of findings for no change of perceptual disorders and dysfunctional eating attitudes and behaviors; n.a: not available.

This mention allowed to inform the methodological strength regarding the COVID-19 specification of the 21 studies using two methodological criteria: measures and data collection. For clarity, a high adequacy corresponds to (a) data collection during the lockdown (e.g., pre-, and post-pandemic, during pandemic, etc.) with (b) measures focused on perceptual disorders and dysfunctional eating attitudes and behaviors during the COVID-19 pandemic. A moderate adequacy corresponds to (a) data collection including lockdown periods with (b) measures focused on the relation between COVID-19 and variables other than perceptual disorders and dysfunctional eating attitudes and behaviors (e.g., Corona Anxiety Scale; Sajedi et al., 2021) or information on the degree of confinement or the duration of confinement (Mannino et al., 2021). A low adequacy corresponds to (a) unclear data collection (b) without any measure specific to COVID-19. Table 3 presents 21 studies with a high COVID-19 adequacy mention: 7 studies were rated “moderate,” 10 studies were rated as “high” and only 4 studies were rated as “low.” All studies with “high” adequacy, including community and (sub-)clinical samples, demonstrated homogenous findings regarding exacerbation of symptoms and maladaptive behaviors, with the exception of Costa et al. (2022) study with mixed findings relating to dysfunctional eating attitudes and behaviors. All studies with “moderate” COVID-19-related adequacy, including community and (sub-)clinical samples, presented homogenous findings in favor of an exacerbation, improvement and no change, with the exception of Baceviciene and Jankauskiene (2021) study whose findings relating to perceptual disturbances are ambiguous. In contrast, studies in low COVID-19 adequacy, including community and (sub-)clinical samples, demonstrated a variability of findings with notable ambiguities in the results relating to perceptual disturbances and dysfunctional eating attitudes and behaviors (Faramarzi et al., 2021). One might conclude that the higher the COVID-19 specification of the evidence, the more the findings point to an exacerbation of perceptual disturbances and dysfunctional eating attitudes and behaviors. Furthermore, notice that studies in (sub-)clinical samples showed unanimous findings of exacerbation and this independently from the level of COVID-19 adequacy. The limited number of studies regarding sub-clinical and clinical populations should limit the generalization of conclusions regarding these populations. However, the results of this review are consistent with conclusions from previous reviews on the exacerbation of perceptual disorders and dysfunctional eating attitudes and behaviors in populations with these health conditions during the COVID-19 pandemic (Miniati et al., 2021; Monteleone et al., 2021; Sideli et al., 2021).

### 3.2. Factors related to perceptual disturbances and dysfunctional eating attitudes and behaviors during the COVID-19 pandemic

Associated to the two main outcomes of the review, two phenomena appear to be associated with perceptual disturbances and dysfunctional eating attitudes and behaviors: social media (including social networks) and physical activity. In the context of stay-at-home orders and self-isolation, results indicated an overall increase of perceptual disturbances and dysfunctional eating attitudes and behaviors linked to media pressure (in terms of promotion of thin/low fat ideals), self-reported time spent on Facebook and maladaptive Facebook use. Moreover, social media seem to have a central role and to mediate the relationship between COVID-19 lockdowns and perceptual disturbance and/or dysfunctional eating attitudes. Fear of others’ perception and social media pressure not only increased perceptual disturbances and dysfunctional eating attitudes and behaviors, but gave rise to a loss of body and eating identity and sense of self, particularly in the LGBTQ + community. Otherwise, the results regarding physical activity and exercise were less congruent because of the conceptual ambivalence demonstrated by some authors (Davis et al., 1997; Hausenblas and Fallon, 2006; Rizk et al., 2015; Danielsen et al., 2018) and catalyzed by the COVID-19 context. On a cautious side, the concept of health, physical activity and exercise refers to habits to maintain a healthy mental state and a healthful occupation or leisure time exercise during the pandemic. On a risk side, the concept of physical exercise, and more particularly excessive exercise, is synonymous, in this review, with inappropriate compensatory behaviors. “Exercise addiction” and “movement fixation” are associated with perceived pressure exercise to avoid weight gain and being sedentary, or it is regarded as an emotional response to COVID-19-related anxiety, fear and depression. In some studies, people developed healthier training habits and diminished their exercise addiction (Baceviciene and Jankauskiene, 2021; Haddad et al., 2021; Trott et al., 2021), and for some others, concerns about physical activity and training duration increased (Robertson et al., 2021; Quathamer and Joy, 2022). Indeed, a lack of structure and disrupted routines, greater exposure to environmental triggers, and difficulties regulating emotions were highlighted as possible precipitants for perceptual disturbances. Among community samples, perceptual disorders and dysfunctional eating attitudes and behaviors were present among different types of populations: “students of both genders,” “women,” “athletes,” “former athletes,” “health club users” (with incongruent results), “the LGBTQ + community” (with incongruent results), “BIPOC,” “overweight women,” the population with orthorexia. White female and heterosexual individuals constitute the majority of the study samples of this review (Schneider et al., 2022). The negative side of COVID-19 relating to perceptual disorders and dysfunctional eating attitudes and behaviors is manifested in the more marginalized and historically and often stigmatized, under-represented populations such as the LGBTQ + community and those who identify as BIPOC (Gullo and Walker, 2021; Brownstone et al., 2022; Schneider et al., 2022). The fact that most investigations were conducted in Western cultures (i.e., Europe or North America) emphasizes the concepts of “perceptual disorders and dysfunctional eating attitudes and behaviors” as culture-bound syndromes which focus on the thin ideal internalization as the/a process of emergence of eating disorders.

### 3.3. Limitations

This review allows a new and up-to-date understanding of the complexity of perceptual disorders and dysfunctional eating attitudes and behaviors related to mental disorder research. However, several limitations, depending on the nature of this literature review, can be noticed and considered to interpret the presented findings. Firstly, as in all reviews, generalization of findings is ascertained because one cannot confirm that no literature was missed. Since the majority of the studies relies on adults’ samples, results may not be generalizable to younger and/or older individuals. Furthermore, the results of this review cannot be generalized to people living with obesity as studies on this population were excluded from this review. Secondly, none of the rigorous double-screening process for quality assessment of all studies, including the use of validated tools was integrated, contrary to Schneider et al.’s systematic review (Schneider et al., 2022). Thirdly, the strength of inclusion of qualitative and quantitative studies to a holistic understanding becomes an interpretative challenge to integrate findings according to mixed-study-design into a meaningful and consistent whole. Regarding the limitation of the studies included in this review, most findings were based on self-report measurements, online convenience sampling, cross-sectional and retrospective designs to establish comparisons or relationships or explanations to pre-pandemic findings, thus questioning the collected high-quality data experiences and cohorts (Pierce et al., 2020). In terms of methodological strength regarding the COVID-19 specification, 4 studies included in this review reported unclear information regarding data collection without any measure specific to COVID-19. Furthermore, only 3 studies on clinical and sub-clinical samples met the inclusion criteria of this review. For this reason, generalization of conclusions of this review to clinical populations should be considered carefully. The lack in the literature about clinical populations highlights the need for more studies that tackle clinical samples to achieve a better and overall understanding of the impact of the COVID-19 pandemic on perceptual disturbances and dysfunctional eating attitudes and behaviors. Finally, the results of the literature search highlighted a predominance of studies conducted in Western cultures and a lack of studies conducted in developing and under-developed countries. This significant lack in the scientific literature indicates that much more studies are needed to provide a more comprehensive pandemic scenario of perceptual disturbances, dysfunctional eating attitudes and behaviors, and general eating disorders features in different countries and populations.

### 3.4. Implications for interventions

This literature review, highlights two main risky scenarios which characterized the COVID-19 period: (World Health Organization, 2020) a plethora of expressions, known and new, that refer to perceptual disturbances in general, sub-clinical and clinical populations (e.g., “negative body image,” “shape and weight concerns,” “thin ideal internalization,” “overvaluation of appearance,” “preoccupation around physical appearance,” “negative body social comparison,” “zoom dysmorphia disorders,” “worsening of body image,” “body dissatisfaction,” “body grief,” “body surveillance”) and (de Medeiros Carvalho et al., 2020) a large spectrum of dysfunctional eating attitudes and behaviors, known and new, in the general population and an extreme aggravation of biopsychological symptoms associated with relapses, in the population with eating disorders (e.g., “emotional eating,” “binge eating,” “restrictive eating,” “disordered eating cognitions,” “preoccupation around food,” “intake fixation,” “uncontrolled eating,” “orthorexia,” “disordered eating thoughts,” “dieting behaviors,” “diet-related stress”). These conclusions tend to raise questions about how to conceive health and a health network designated to assess and intervene during critical life events. There is an urgent need of conceiving interventions even in the aftermath of lockdowns periods since studies suggests that dysfunctional eating behaviors, such as emotional and uncontrolled eating could be part of an entrenched maladaptive pattern of coping strategies, which could exacerbate during intense times of distress (Coulthard et al., 2021).

**Figure 3:**
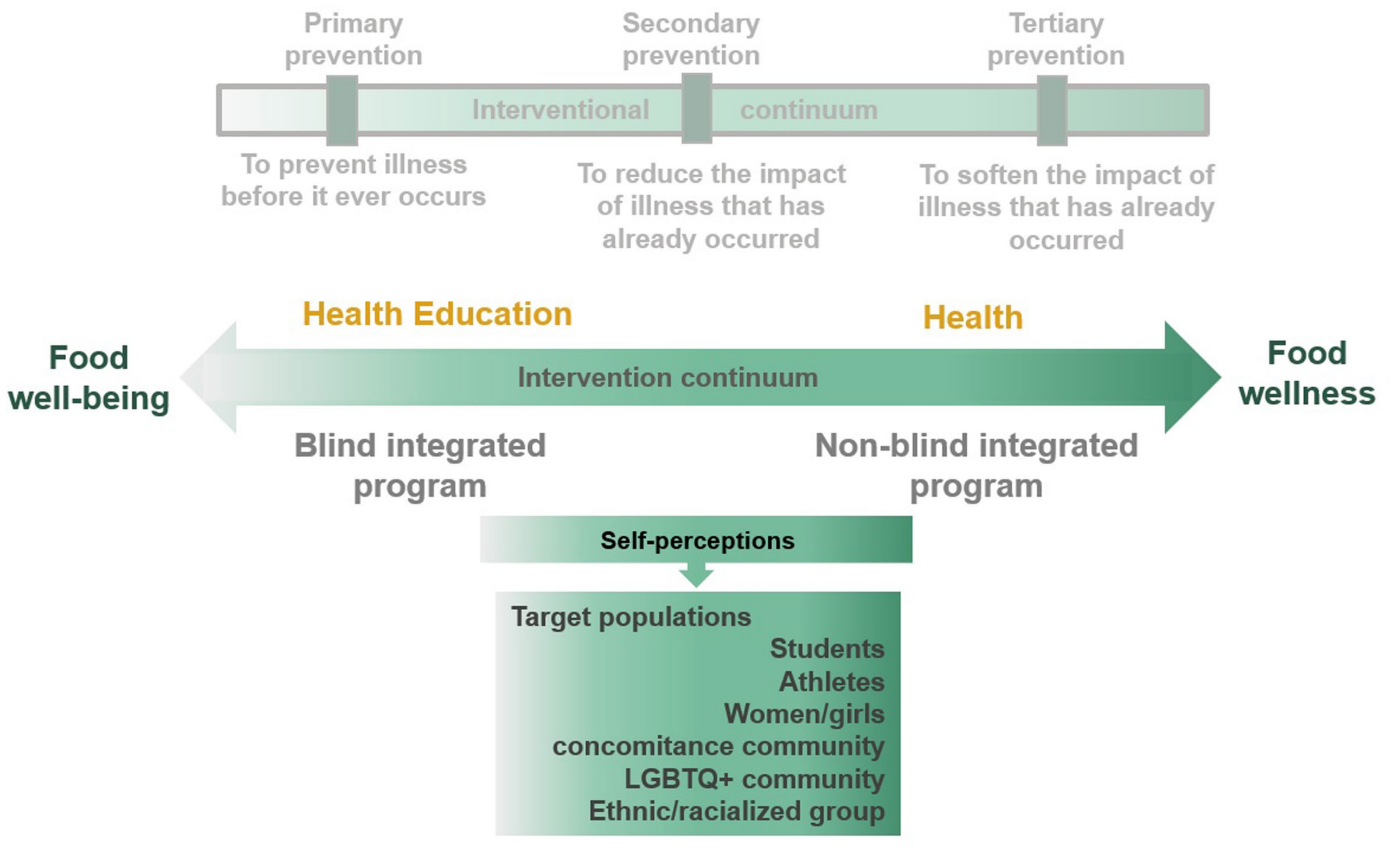
An intervention continuum suitable to the dimensional perspective of dysfunctional eating attitudes and behaviors.

Historically, “intervention” in mental health corresponds to the famous trichotomy: primary, secondary, and tertiary prevention. The main expected outcome of a health intervention is a “state of complete physical, mental and social well-being and not merely the absence of disease or infirmity” (World Health Organization, 1948). Primary prevention impedes illness before it ever occurs, whereas secondary and tertiary preventions reduce or soften the impact of illness that has already occurred. In other words, primary prevention gathers educational programs about healthy and safe habits (including eating well and exercising regularly). Secondary and tertiary preventions focus on therapeutic and rehabilitation programs equipped with personal strategies to prevent chronic illness and strategies for living well. As mentioned previously, the situation of the COVID-19 pandemic impacts both the general population with disordered eating (primary prevention category) and the sub-clinical and clinical populations with eating disorders (secondary and tertiary prevention categories) that are heading towards perceptual disorders. With regards to the conclusion of this review, Figure 3 presents a conceptualization of an intervention continuum suitable to the dimensional perspective of dysfunctional eating attitudes and behaviors.

Future interventions could fluctuate along an intervention continuum, between a healthy diet education and food well-being focused on intuitive eating at one end of the continuum, and food wellness focus on self-compassion/self-acceptance at the other end of the pole. All along the continuum, self-perceptions (e.g., promotion of positive body image, body image flexibility over time, body-food choice congruence as protective factors) would be the target of intervention programs on (dysfunctional) eating attitudes and behaviors focusing on different populations (i.e., students, athletes, women/girls, concomitance communities such as overweight women with eating disorders, the LGBTQ + community and ethnic/racialized groups). As opposed to the three categories of intervention based on a purely medical model of health, this bipolar intervention continuum attempts to respond to the reality of food wellness springing from perceptual disorders and dysfunctional eating attitudes and behavior outcomes of the present and previous reviews. As presented in Figure 2, the dichotomy between health education programs promoting food well-being and health programs focusing on food wellness depends on the importance of having a hidden goal in general population (i.e., not revealing the program objectives to the target population; Monthuy-Blanc et al., 2020). The need to resort to a hidden objective in health education came from conflicting messages from obesity-only programs and eating disorders-only programs (Leme et al., 2019) or the risk to be fascinating by alarmist message about complications of eating disorders (e.g., induced vomiting; Stice and Shaw, 2004; Noordenbos, 2016; Monthuy-Blanc et al., 2020).

## 4. Conclusion

In conclusion, findings of the current literature review complement and extend the results from previous reviews—systematic, narrative, and systematic scoping—conducted on eating disorders, disordered eating, and body image during the COVID-19 pandemic (Ahuja and Banerjee, 2021; Linardon et al., 2021; Miniati et al., 2021; Monteleone et al., 2021; Sideli et al., 2021; Devoe et al., 2022; Gao et al., 2022; Haghshomar et al., 2022; Savarese et al., 2022; Schafer et al., 2022; Schneider et al., 2022). Previously published reviews explored research spanning the period from January 2020 to October 2021. To the authors’ knowledge, this is the first review covering studies published 2 years after the COVID-19 outbreak. Furthermore, the current review provides a comprehensive depiction of perceptual disturbances and dysfunctional eating attitudes and behaviors in the general, sub-clinical and clinical populations during the pandemic crisis. If there is currently high variability in study designs, measures used, participant samples and the way to integrate the COVID-19 particularity (called COVID-19 adequacy) across different studies, this review highlights multiple notable considerations for future research and reflection.

## Author contributions

JM–B, GC, and SA created and conceptualized the study, led the review, analyzed and interpreted the data, and critically revised the manuscript and provided constructive comments. JM–B and GC wrote the first draft. All authors contributed to the article and approved the submitted version.

## Funding

This work was supported by the settlement fund of the Fondation de l’Institut Universitaire de santé mentale de Montréal affilié au CR-IUSMM, the RBC Royal Bank Foundation, the Takeda Canada Foundation, 2021–2024 awarded to the first author (JM-B); and a postdoctoral grant awarded to GC by the Fonds de Recherche du Québec—Santé (FRQS), and the Fondation de l’Institut Universitaire de santé mentale de Montréal affilié au CR-IUSMM.

## Conflict of interest

The authors declare that the research was conducted in the absence of any commercial or financial relationships that could be construed as a potential conflict of interest.

## Publisher’s note

All claims expressed in this article are solely those of the authors and do not necessarily represent those of their affiliated organizations, or those of the publisher, the editors and the reviewers. Any product that may be evaluated in this article, or claim that may be made by its manufacturer, is not guaranteed or endorsed by the publisher.

## References

[ref1] Ahuja K. K. Banerjee D. A. (2021). Psychosocial exploration of body dissatisfaction: a narrative review with a focus on India during COVID-19. Front. Glob. Women's Health 2:669013. doi: 10.3389/fgwh.2021.669013, PMID: 34816220 PMC8593986

[ref2] Alvarenga M. S. Pereira R. F. Scagliusi F. B. Philippi S. T. Estima C. C. Croll J. (2010). Psychometric evaluation of the disordered eating attitude scale (DEAS) English version. Appetite 55, 374–376. doi: 10.1016/j.appet.2010.07.003, PMID: 20624435

[ref3] American Psychiatric Association. Diagnostic and Statistical Manual Of Mental Disorders (DSM-V). Arlington: American Psychiatric Association (2013).

[ref4] Andersen N. Swami V. (2021). Science mapping research on body image: a bibliometric review of publications in body image, 2004–2020. Body Image 38, 106–119. doi: 10.1016/j.bodyim.2021.03.015, PMID: 33838539

[ref5] Baceviciene M. Jankauskiene R. (2021). Changes in sociocultural attitudes towards appearance, body image, eating attitudes and behaviors, physical activity, and quality of life in students before and during COVID-19 lockdown. Appetite 166:105452. doi: 10.1016/j.appet.2021.105452, PMID: 34107292 PMC9756094

[ref6] Brown S. Opitz M. C. Peebles A. I. Sharpe H. Duffy F. Newman E. (2021). A qualitative exploration of the impact of COVID-19 on individuals with eating disorders in the UK. Appetite 156:104977. doi: 10.1016/j.appet.2020.104977, PMID: 32991945 PMC7521890

[ref7] Brownstone L. M. Greene A. K. Kelly D. A. Maloul E. K. Norling H. N. Rockholm R. H.. (2022). "are people thinking I'm a vector…because I'm fat?": cisgender experiences of body, eating, and identity during COVID-19. Body Image 40, 256–266. doi: 10.1016/j.bodyim.2022.01.002, PMID: 35077950 PMC8783103

[ref8] Bruch H. (1962). Perceptual and conceptual disturbances in anorexia nervosa. Psychosom. Med. 24, 187–194. doi: 10.1097/00006842-196203000-00009, PMID: 13873828

[ref9] Bruch H. (1974). Perils of behavior modification in treatment of anorexia nervosa. JAMA 230, 1419–1422. doi: 10.1001/jama.230.10.1419, PMID: 4479645

[ref10] Buckley G. L. Hall L. E. Lassemillante A. M. Belski R. (2021). Disordered eating & body image of current and former athletes in a pandemic; a convergent mixed methods study—what can we learn from COVID-19 to support athletes through transitions? J. Eat. Disord. 9:73. doi: 10.1186/s40337-021-00427-3, PMID: 34167589 PMC8223527

[ref11] Burrows A. Cooper M. (2002). Possible risk factors in the development of eating disorders in overweight pre-adolescent girls. Int. J. Obes. Relat. Metab. Disord. 26, 1268–1273. doi: 10.1038/sj.ijo.0802033, PMID: 12187406

[ref12] Cash T. F. Deagle E. A. III (1997). The nature and extent of body-image disturbances in anorexia nervosa and bulimia nervosa: a meta-analysis. Int. J. Eat. Disord. 22, 107–126. doi: 10.1002/(SICI)1098-108X(199709)22:2<107::AID-EAT1>3.0.CO;2-J, PMID: 9261648

[ref13] Cooper M. Reilly E. E. Siegel J. A. Coniglio K. Sadeh-Sharvit S. Pisetsky E. M.. (2022). Eating disorders during the COVID-19 pandemic and quarantine: an overview of risks and recommendations for treatment and early intervention. Eat. Disord. 30, 54–76. doi: 10.1080/10640266.2020.1790271, PMID: 32644868 PMC7929530

[ref14] Corno G. Paquette A. Monthuy-Blanc J. Ouellet M. Bouchard S. (2022). The relationship between Women's negative body image and disordered eating behaviors during the COVID-19 pandemic: a cross-sectional study. Front. Psychol. 13:856933. doi: 10.3389/fpsyg.2022.856933, PMID: 35401386 PMC8987766

[ref15] Costa M. L. Costa M. de Souza M. da Silva D. G. Dos Santos Vieira D. A. Mendes-Netto R. S. (2022). Cognitive restraint, emotional eating and uncontrolled eating: exploring factors associated with the cycle of behaviors during the COVID-19 pandemic. Food Qual. Prefer. 100:104579. doi: 10.1016/j.foodqual.2022.104579, PMID: 35280669 PMC8905886

[ref16] Coulthard H. Sharps M. Cunliffe L. van den Tol A. (2021). Eating in the lockdown during the Covid 19 pandemic; self-reported changes in eating behaviour, and associations with BMI, eating style, coping and health anxiety. Appetite 161:105082. doi: 10.1016/j.appet.2020.105082, PMID: 33476651 PMC7976455

[ref17] Czepczor-Bernat K. Modrzejewska J. Modrzejewska A. Matusik P. (2021). Do COVID-19-related stress, being overweight, and body dissatisfaction contribute to more disordered eating in polish women?—a cluster analysis approach. Int. J. Environ. Res. Public Health 18:13100. doi: 10.3390/ijerph182413100, PMID: 34948710 PMC8701286

[ref18] Danielsen M. Rø Ø. Bjørnelv S. (2018). How to integrate physical activity and exercise approaches into inpatient treatment for eating disorders: fifteen years of clinical experience and research. J. Eat. Disord. 6:34. doi: 10.1186/s40337-018-0203-5, PMID: 30258631 PMC6154924

[ref19] Davis C. Katzman D. K. Kaptein S. Kirsh C. Brewer H. Kalmbach K.. (1997). The prevalence of high-level exercise in the eating disorders: etiological implications. Compr. Psychiatry 38, 321–326. doi: 10.1016/s0010-440x(97)90927-5, PMID: 9406737

[ref20] de Medeiros Carvalho P. M. Moreira M. M. de Oliveira M. N. A. Landim J. M. M. Neto M. L. R. (2020). The psychiatric impact of the novel coronavirus outbreak. Psychiatry Res. 286:112902. doi: 10.1016/j.psychres.2020.112902, PMID: 32146248 PMC7133679

[ref21] Devoe J. D. Han A. Anderson A. Katzman D. K. Patten S. B. Soumbasis A.. (2022). The impact of the COVID-19 pandemic on eating disorders: a systematic review. Int. J. Eat. Disord. 56, 5–25. doi: 10.1002/eat.23704 35384016 PMC9087369

[ref22] di Renzo L. Gualtieri P. Cinelli G. Bigioni G. Soldati L. Attinà A.. (2020). Psychological aspects and eating habits during COVID-19 home confinement: results of EHLC-COVID-19 Italian online survey. Nutrients 12:2152. doi: 10.3390/nu12072152, PMID: 32707724 PMC7401000

[ref23] Fairburn C. G. Cooper Z. Shafran R. (2003). Cognitive behaviour therapy for eating disorders: a “transdiagnostic” theory and treatment. Behav. Res. Ther. 41, 509–528. doi: 10.1016/s0005-7967(02)00088-8, PMID: 12711261

[ref24] Faramarzi M. Mardaniyan Ghahfarrokhi M. Hemati Farsani Z. Raisi Z. Jamali M. Baker J. S. (2021). The relationship between physical activity, body image, and eating disorders during the COVID-19 pandemic in high-school girls. Int. J. Epidemiol. Res. 8, 152–159. doi: 10.34172/IJER.2021.28

[ref25] Fernández-Aranda F. Casas M. Claes L. Bryan D. C. Favaro A. Granero R.. (2020). COVID-19 and implications for eating disorders. Eur. Eat. Disord. Rev. 28, 239–245. doi: 10.1002/erv.2738, PMID: 32346977 PMC7267370

[ref26] Ferrell E. L. Watford T. S. Braden A. (2020). Emotion regulation difficulties and impaired working memory interact to predict boredom emotional eating. Appetite 144:104450. doi: 10.1016/j.appet.2019.104450, PMID: 31525419

[ref27] Flaudias V. Iceta S. Zerhouni O. Rodgers R. F. Billieux J. Llorca P. M.. (2020). COVID-19 pandemic lockdown and problematic eating behaviors in a student population. J. Behav. Addict. 9, 826–835. doi: 10.1556/2006.2020.00053, PMID: 32976112 PMC8943668

[ref28] Gao Y. Bagheri N. Furuya-Kanamori L. (2022). Has the COVID-19 pandemic lockdown worsened eating disorders symptoms among patients with eating disorders? A systematic review. J. Public Health 30, 2743–2752. doi: 10.1007/s10389-022-01704-4, PMID: 35369670 PMC8961480

[ref29] Glashouwer K. A. van der Veer R. M. Adipatria F. de Jong P. J. Vocks S. (2019). The role of body image disturbance in the onset, maintenance, and relapse of anorexia nervosa: a systematic review. Clin. Psychol. Rev. 74:101771. doi: 10.1016/j.cpr.2019.101771, PMID: 31751876

[ref30] Gobin K. C. Mills J. S. McComb S. E. (2021). The effects of the COVID-19 pandemic lockdown on eating, body image, and social media habits among women with and without symptoms of orthorexia nervosa. Front. Psychol. 12:716998. doi: 10.3389/fpsyg.2021.716998, PMID: 34975611 PMC8714632

[ref31] Gullo N. Walker D. C. (2021). Increased videoconferencing after COVID-19 stay-at-home orders increased depression and anxiety but did not impact appearance satisfaction or binge eating. Comput. Hum. Behav. 3:100080. doi: 10.1016/j.chbr.2021.100080

[ref32] Haddad C. Zakhour M. Bou kheir M. Haddad R. al Hachach M. Sacre H.. (2020). Association between eating behavior and quarantine/confinement stressors during the coronavirus disease 2019 outbreak. J. Eat. Disord. 8:40. doi: 10.1186/s40337-020-00317-0, PMID: 32879730 PMC7458649

[ref33] Haddad C. Zakhour M. Siddik G. Haddad R. Sacre H. Salameh P. (2021). COVID-19 outbreak: does confinement have any impact on weight change perception? Nutr. Clin. Métab. 35, 137–143. doi: 10.1016/j.nupar.2021.02.003

[ref34] Haghshomar M. Shobeiri P. Brand S. Rossell S. L. Akhavan Malayeri A. Rezaei N. (2022). Changes of symptoms of eating disorders (ED) and their related psychological health issues during the COVID-19 pandemic: a systematic review and meta-analysis. J. Eat. Disord. 10:51. doi: 10.1186/s40337-022-00550-9, PMID: 35418108 PMC9006500

[ref35] Hausenblas H. A. Fallon E. A. (2006). Exercise and body image: a meta-analysis. Psychol. Health 21, 33–47. doi: 10.1080/14768320500105270

[ref36] Jackson A. M. Parker L. Sano Y. Cox A. E. Lanigan J. (2022). Associations between body image, eating behavior, and diet quality. Nutr. Health:2601060221090696. doi: 10.1177/02601060221090696, PMID: 35369805

[ref37] Khan M. A. Smith J. E. M. (2020). “Covibesity,” a new pandemic. Obes Med 19:100282. doi: 10.1016/j.obmed.2020.100282, PMID: 32835125 PMC7371584

[ref38] Koven N. S. Abry A. W. (2015). The clinical basis of orthorexia nervosa: emerging perspectives. Neuropsychiatr. Dis. Treat. 11, 385–394. doi: 10.2147/NDT.S61665, PMID: 25733839 PMC4340368

[ref39] Leme A. C. B. Philippi S. T. Thompson D. Nicklas T. Baranowski T. (2019). “Healthy habits, healthy girls—Brazil”: an obesity prevention program with added focus on eating disorders. Eat. Weight Disord. 24, 107–119. doi: 10.1007/s40519-018-0510-5, PMID: 29730727

[ref40] Leung F. Schwartzman A. Steiger H. (1996). Testing a dual-process family model in understanding the development of eating pathology: a structural equation modeling analysis. Int. J. Eat. Disord. 20, 367–375. doi: 10.1002/(SICI)1098-108X(199612)20:4<367::AID-EAT4>3.0.CO;2-L, PMID: 8953324

[ref41] Levine M. P. Piran N. (2004). The role of body image in the prevention of eating disorders. Body Image 1, 57–70. doi: 10.1016/S1740-1445(03)00006-8 18089141

[ref42] Linardon J. Tylka T. L. Fuller-Tyszkiewicz M. (2021). Intuitive eating and its psychological correlates: a meta-analysis. Int. J. Eat. Disord. 54, 1073–1098. doi: 10.1002/eat.23509, PMID: 33786858

[ref43] Lucibello K. M. Vani M. F. Koulanova A. DeJonge M. L. Ashdown-Franks G. Sabiston C. M. (2021). # quarantine15: a content analysis of Instagram posts during COVID-19. Body Image 38, 148–156. doi: 10.1016/j.bodyim.2021.04.002, PMID: 33892438 PMC9760216

[ref44] Madalı B. Alkan Ş. B. Örs E. D. Ayrancı M. Taşkın H. Kara H. H. (2021). Emotional eating behaviors during the COVID-19 pandemic: a cross-sectional study. Clin. Nutr. ESPEN 46, 264–270. doi: 10.1016/j.clnesp.2021.09.745, PMID: 34857207 PMC8492000

[ref45] Mannino G. Salerno L. Bonfanti R. C. Albano G. Lo C. G. (2021). The impact of Facebook use on self-reported eating disorders during the COVID-19 lockdown. BMC Psychiatr. 21:611. doi: 10.1186/s12888-021-03628-x, PMID: 34876064 PMC8651245

[ref46] Miniati M. Marzetti F. Palagini L. Marazziti D. Orrù G. Conversano C.. (2021). Eating disorders spectrum during the COVID pandemic: a systematic review. Front. Psychol. 12:663376. doi: 10.3389/fpsyg.2021.663376, PMID: 34658992 PMC8511307

[ref47] Miskovic-Wheatley J. Koreshe E. Kim M. Simeone R. Maguire S. (2022). The impact of the COVID-19 pandemic and associated public health response on people with eating disorder symptomatology: an Australian study. J. Eat. Disord. 10:9. doi: 10.1186/s40337-021-00527-0, PMID: 35039076 PMC8762631

[ref48] Monteleone A. M. Cascino G. Barone E. Carfagno M. Monteleone P. (2021). COVID-19 pandemic and eating disorders: what can we learn about psychopathology and treatment? A systematic review. Curr. Psychiatr. Rep. 23:83. doi: 10.1007/s11920-021-01294-0, PMID: 34674064 PMC8528944

[ref49] Monthuy-Blanc J. Bouchard S. Ouellet M. Corno G. Iceta S. Rousseau M. (2020). “E LoriCorps immersive body rating scale”: exploring the assessment of body image disturbances from Allocentric and egocentric perspectives. J. Clin. Med. 9:2926. doi: 10.3390/jcm9092926, PMID: 32927847 PMC7564525

[ref50] Monthuy-Blanc J. Corno G. Bouchard S. St-Pierre M. J. Bourbeau F. Mostefa-Kara L.. (2022). Body perceptions, occupations, eating attitudes, and behaviors emerged during the pandemic: an exploratory cluster analysis of eaters profiles. Front. Psychol. 13:949373. doi: 10.3389/fpsyg.2022.949373, PMID: 36544438 PMC9762356

[ref51] Monthuy-Blanc J. Lemieux V. Thériault J. Rousseau M. (2020). Exploratory study: a blind integrated school-based prevention program on eating disorders and obesity. Can. J. Commun. Ment. Health 39, 61–84. doi: 10.7870/cjcmh-2020-027

[ref52] Neumark-Sztainer D. Wall M. M. Story M. Perry C. L. (2003). Correlates of unhealthy weight-control behaviors among adolescents: implications for prevention programs. Health Psychol. 22, 88–98. doi: 10.1037//0278-6133.22.1.88, PMID: 12558206

[ref53] Nisticò V. Bertelli S. Tedesco R. Anselmetti S. Priori A. Gambini O.. (2021). The psychological impact of COVID-19-related lockdown measures among a sample of Italian patients with eating disorders: a preliminary longitudinal study. Eat. Weight Disord. 26, 2771–2777. doi: 10.1007/s40519-021-01137-0, PMID: 33582970 PMC7882047

[ref54] Noordenbos G. (2016). How to block the ways to eating disorders. Eat. Disord. 24, 47–53. doi: 10.1080/10640266.2015.1113827, PMID: 26643364

[ref55] Pearl R. L. (2020). Weight stigma and the “Quarantine-15”. Obesity (Silver Spring, Md.) 28, 1180–1181. doi: 10.1002/oby.22850 32324954 PMC7264559

[ref56] Pierce M. McManus S. Jessop C. John A. Hotopf M. Ford T.. (2020). Says who? The significance of sampling in mental health surveys during COVID-19. Lancet Psychiatry 7, 567–568. doi: 10.1016/S2215-0366(20)30237-6, PMID: 32502467 PMC7266586

[ref57] Pikoos T. D. Buzwell S. Sharp G. Rossell S. L. (2021). The zoom effect: exploring the impact of video calling on appearance dissatisfaction and interest in aesthetic treatment during the COVID-19 pandemic. Aesthet. Surg. J. 41, NP2066–NP2075. doi: 10.1093/asj/sjab257, PMID: 34146086

[ref58] Pineda-García G. Serrano-Medina A. Ochoa-Ruíz E. Martínez A. L. (2021). Body image, anxiety, and bulimic behavior during confinement due to COVID-19 in Mexico. Healthcare (Basel) 9:1435. doi: 10.3390/healthcare9111435, PMID: 34828482 PMC8618812

[ref59] Pino O. (2022). Is zoom dysmorphia a new disorder? Acta Biomed 92:e2021303. doi: 10.23750/abm.v92i6.12618, PMID: 35075054 PMC8823569

[ref60] Quathamer N. Joy P. (2022). Being in a queer time: exploring the influence of the COVID-19 pandemic on LGBTQ+ body image. Nutr. Diet. 79, 400–410. doi: 10.1111/1747-0080.12699, PMID: 34337841 PMC8441880

[ref61] Rizk M. Lalanne C. Berthoz S. Kern L. Group E. Godart N. (2015). Problematic exercise in anorexia nervosa: testing potential risk factors against different definitions. PLoS One 10:e0143352. doi: 10.1371/journal.pone.0143352, PMID: 26618359 PMC4664470

[ref62] Robertson M. Duffy F. Newman E. Bravo C. P. Ates H. H. Sharpe H. (2021). Exploring changes in body image, eating and exercise during the COVID-19 lockdown: a UK survey. Appetite 159:105062. doi: 10.1016/j.appet.2020.105062, PMID: 33278549 PMC7711175

[ref63] Rodgers R. F. Lombardo C. Cerolini S. Franko D. L. Omori M. Fuller-Tyszkiewicz M.. (2020). The impact of the COVID-19 pandemic on eating disorder risk and symptoms. Int. J. Eat. Disord. 53, 1166–1170. doi: 10.1002/eat.23318, PMID: 32476175 PMC7300468

[ref64] Sajedi H. Kirkbir F. Bayram M. Karatas B. (2021). The impairment of body image of the students of Agri Ibrahim Çeçen University Faculty of Sports Sciences and the effect of the anxiety of caught Corona disease on their nutrition habits. Shanlax Int. J. Educ. 9, 52–58. doi: 10.34293/education.v9iS2-Sep.4370

[ref65] Salari N. Hosseinian-Far A. Jalali R. Vaisi-Raygani A. Rasoulpoor S. Mohammadi M.. (2020). Prevalence of stress, anxiety, depression among the general population during the COVID-19 pandemic: a systematic review and meta-analysis. Glob. Health 16:57. doi: 10.1186/s12992-020-00589-w, PMID: 32631403 PMC7338126

[ref66] Sanlier N. Kocabas Ş. Ulusoy H. G. Celik B. (2022). The relationship between adults’ perceptions, attitudes of COVID-19, intuitive eating, and mindful eating behaviors. Ecol. Food Nutr. 61, 90–109. doi: 10.1080/03670244.2021.1968849, PMID: 34435919

[ref67] Savarese G. Milano W. D. Carpinelli L. (2022). Eating disorders (EDs) and the COVID-19 pandemic: a pilot study on the impact of phase II of the lockdown. BioMed 2, 110–116. doi: 10.3390/biomed2010012

[ref68] Scarmozzino F. Visioli F. (2020). Covid-19 and the subsequent lockdown modified dietary habits of almost half the population in an Italian sample. Foods 9:675. doi: 10.3390/foods9050675, PMID: 32466106 PMC7278864

[ref69] Schafer K. M. Lieberman A. Sever A. C. Joiner T. (2022). Prevalence rates of anxiety, depressive, and eating pathology symptoms between the pre- and peri-COVID-19 eras: a meta-analysis. J. Affect. Disord. 298, 364–372. doi: 10.1016/j.jad.2021.10.115, PMID: 34740748 PMC8593520

[ref70] Schlegl S. Maier J. Meule A. Voderholzer U. (2020). Eating disorders in times of the COVID-19 pandemic—results from an online survey of patients with anorexia nervosa. Int. J. Eat. Disord. 53, 1791–1800. doi: 10.1002/eat.23374, PMID: 32841413 PMC7461418

[ref71] Schmid J. Rose K. Hadler N. Amaro X. Frank A. Wilkie E.. (2022). Content analysis of the impact of COVID-19 on weight and shape control behaviors and social media content of U.S. adolescents and young adults. Eat. Behav. 45:101635. doi: 10.1016/j.eatbeh.2022.101635, PMID: 35567879 PMC9074298

[ref72] Schneider J. Pegram G. Gibson B. Talamonti D. Tinoco A. Craddock N.. (2022). A mixed-studies systematic review of the experiences of body image, disordered eating, and eating disorders during the COVID-19 pandemic. Int. J. Eat. Disord. 56, 26–67. doi: 10.1002/eat.23706, PMID: 35322449 PMC9087368

[ref73] Sideli L. Lo Coco G. Bonfanti R. C. Borsarini B. Fortunato L. Sechi C.. (2021). Effects of COVID-19 lockdown on eating disorders and obesity: a systematic review and meta-analysis. Eur. Eat. Disord. Rev. 29, 826–841. doi: 10.1002/erv.2861, PMID: 34460991 PMC8652707

[ref74] Stice E. (2002). Risk and maintenance factors for eating pathology: a meta-analytic review. Psychol. Bull. 128, 825–848. doi: 10.1037/0033-2909.128.5.825, PMID: 12206196

[ref75] Stice E. (2022). A prospective test of the dual-pathway model of bulimic pathology: mediating effects of dieting and negative affect. J. Abnorm. Psychol. 110, 124–135. doi: 10.1037//0021-843x.110.1.124, PMID: 11261386

[ref76] Stice E. Mazotti L. Krebs M. Martin S. (1998). Predictors of adolescent dieting behaviors: a longitudinal study. Psychol. Addict. Behav. 12, 195–205. doi: 10.1037/0893-164X.12.3.195

[ref77] Stice E. Nemeroff C. Shaw H. E. (1996). Test of the dual pathway model of bulimia nervosa: evidence for dietary restraint and affect regulation mechanisms. J. Soc. Clin. Psychol. 15, 340–363. doi: 10.1521/jscp.1996.15.3.340

[ref78] Stice E. Shaw H. (2004). Eating disorder prevention programs: a meta-analytic review. Psychol. Bull. 130, 206–227. doi: 10.1037/0033-2909.130.2.206, PMID: 14979770

[ref79] Sundgot-Borgen J. Torstveit M. (2010). Aspects of disordered eating continuum in elite high-intensity sports. Scand. J. Med. Sci. Sports 20, 112–121. doi: 10.1111/j.1600-0838.2010.01190.x, PMID: 20840569

[ref80] Swami V. Horne G. Furnham A. (2021). COVID-19-related stress and anxiety are associated with negative body image in adults from the United Kingdom. Pers. Individ. Dif. 170:110426. doi: 10.1016/j.paid.2020.110426, PMID: 33046945 PMC7539826

[ref81] Termorshuizen J. D. Watson H. J. Thornton L. M. Borg S. Flatt R. E. MacDermod C. M.. (2020). Early impact ofCOVID‐19 on individuals withself‐reportedeating disorders: a survey of ~1,000 individuals in the United States and the Netherlands. Int. J. Eat. Disord. 53, 1780–1790. doi: 10.1002/eat.23353, PMID: 32720399

[ref82] Todisco P. Donini L. M. (2021). Eating disorders and obesity (ED&O) in the COVID-19 storm. Eat. Weight Disord. 26, 747–750. doi: 10.1007/s40519-020-00938-z, PMID: 32488728 PMC7265870

[ref83] Torales J. O’Higgins M. Castaldelli-Maia J. M. Ventriglio A. (2020). The outbreak of COVID-19 coronavirus and its impact on global mental health. Int. J. Soc. Psychiatry 66, 317–320. doi: 10.1177/0020764020915212 32233719

[ref84] Touyz S. Lacey H. Hay P. (2020). Eating disorders in the time of COVID-19. J. Eat. Disord. 8, 19–13. doi: 10.1186/s40337-020-00295-3, PMID: 32337045 PMC7170399

[ref85] Tribole E. Resch E.. Intuitive Eating: A Revolutionary Anti-diet Approach. New York, NY: St. Martin's Essentials (2020).

[ref86] Trott M. Johnstone J. Pardhan S. Barnett Y. Smith L. (2021). Changes in body dysmorphic disorder, eating disorder, and exercise addiction symptomology during the COVID-19 pandemic: a longitudinal study of 319 health club users. Psychiatry Res. 298:113831. doi: 10.1016/j.psychres.2021.113831, PMID: 33652248 PMC9754709

[ref87] Turgeon M. È. Meilleur D. Blondin S. (2015). Évaluation des attitudes et des comportements alimentaires: comparaison entre un groupe d’adolescentes athlètes pratiquant un sport esthétique et un groupe témoin. Neuropsychiat. Enfance Adolesc. 63, 175–182. doi: 10.1016/j.neurenf.2015.01.001

[ref88] Vall-Roqué H. Andrés A. Saldana C. (2021). The impact of COVID-19 lockdown on social network sites use, body image disturbances and self-esteem among adolescent and young women. Prog. Neuro-Psychopharmacol. Biol. Psychiatry 110:110293. doi: 10.1016/j.pnpbp.2021.110293, PMID: 33662532 PMC8569938

[ref89] Vindegaard N. Benros M. E. (2020). COVID-19 pandemic and mental health consequences: systematic review of the current evidence. Brain Behav. Immun. 89, 531–542. doi: 10.1016/j.bbi.2020.05.048, PMID: 32485289 PMC7260522

[ref90] Vuillier L. May L. Greville-Harris M. Surman R. Moseley R. L. (2021). The impact of the COVID-19 pandemic on individuals with eating disorders: the role of emotion regulation and exploration of online treatment experiences. J. Eat. Disord. 9, 10–18. doi: 10.1186/s40337-020-00362-9, PMID: 33436064 PMC7802411

[ref91] Weissman R. S. Bauer S. Thomas J. J. (2020). Access to evidence-based care for eating disorders during the COVID-19 crisis. Int. J. Eat. Disord. 53, 639–646. doi: 10.1002/eat.23279, PMID: 32338400 PMC7267278

[ref92] Weissman R. S. Hay P. (2022). People's lived experience with an eating disorder during the COVID-19 pandemic: a joint virtual issue of research published in leading eating disorder journals. Int. J. Eat. Disord. 55, 155–160. doi: 10.1002/eat.23653, PMID: 35099825 PMC9015291

[ref93] World Health Organization. Constitution of the World Health Organization. Geneva: World Health Organization (1948).

[ref94] World Health Organization. (2020). WHO Director-General’s opening remarks at the media briefing on COVID-19—11 2020. WHO Director-General’s speeches. Available at: https://www.who.int/director-general/speeches/detail/who-director-general-s-opening-remarks-at-the-media-briefing-on-covid-19---11-march-2020 [Accessed October 31, 2022].

[ref95] Zhang J. Lu H. Zeng H. Zhang S. du Q. Jiang T.. (2020). The differential psychological distress of populations affected by the COVID-19 pandemic. Brain Behav. Immun. 87, 49–50. doi: 10.1016/j.bbi.2020.04.031, PMID: 32304883 PMC7156946

[ref96] Zhou Y. Wade T. D. (2021). The impact of COVID-19 on body-dissatisfied female university students. Int. J. Eat. Disord. 54, 1283–1288. doi: 10.1002/eat.23521, PMID: 33851442 PMC8250175

